# Unraveling the Potential Role of NEDD4-like E3 Ligases in Cancer

**DOI:** 10.3390/ijms232012380

**Published:** 2022-10-16

**Authors:** Sujitha Jayaprakash, Mangala Hegde, Bandari BharathwajChetty, Sosmitha Girisa, Mohammed S. Alqahtani, Mohamed Abbas, Gautam Sethi, Ajaikumar B. Kunnumakkara

**Affiliations:** 1 Cancer Biology Laboratory, Department of Biosciences and Bioengineering, Indian Institute of Technology (IIT) Guwahati, Guwahati 781039, Assam, India; 2 Radiological Sciences Department, College of Applied Medical Sciences, King Khalid University, Abha 61421, Saudi Arabia; 3 BioImaging Unit, Space Research Centre, Michael Atiyah Building, University of Leicester, Leicester LE1 7RH, UK; 4 Electrical Engineering Department, College of Engineering, King Khalid University, Abha 61421, Saudi Arabia; 5 Electronics and Communications Department, College of Engineering, Delta University for Science and Technology, Gamasa 35712, Egypt; 6 Department of Pharmacology, Yong Loo Lin School of Medicine, National University of Singapore, Singapore 117600, Singapore

**Keywords:** cancer, ubiquitination, NEDD4, E3 ligases, tumor suppressor, oncogene, targeted therapy

## Abstract

Cancer is a deadly disease worldwide, with an anticipated 19.3 million new cases and 10.0 million deaths occurring in 2020 according to GLOBOCAN 2020. It is well established that carcinogenesis and cancer development are strongly linked to genetic changes and post-translational modifications (PTMs). An important PTM process, ubiquitination, regulates every aspect of cellular activity, and the crucial enzymes in the ubiquitination process are E3 ubiquitin ligases (E3s) that affect substrate specificity and must therefore be carefully regulated. A surfeit of studies suggests that, among the E3 ubiquitin ligases, neuronal precursor cell-expressed developmentally downregulated 4 (NEDD4)/NEDD4-like E3 ligases show key functions in cellular processes by controlling subsequent protein degradation and substrate ubiquitination. In addition, it was demonstrated that NEDD4 mainly acts as an oncogene in various cancers, but also plays a tumor-suppressive role in some cancers. In this review, to comprehend the proper function of NEDD4 in cancer development, we summarize its function, both its tumor-suppressive and oncogenic role, in multiple types of malignancies. Moreover, we briefly explain the role of NEDD4 in carcinogenesis and progression, including cell survival, cell proliferation, autophagy, cell migration, invasion, metastasis, epithelial-mesenchymal transition (EMT), chemoresistance, and multiple signaling pathways. In addition, we briefly explain the significance of NEDD4 as a possible target for cancer treatment. Therefore, we conclude that targeting NEDD4 as a therapeutic method for treating human tumors could be a practical possibility.

## 1. Introduction

Cancer is one of the top causes of death, which accounts for 19,292,789 new cases and 9,958,133 deaths worldwide according to the GLOBOCAN 2020 [1,2,3]. It is a deadly disease, and it is believed that human beings have been inflicted by cancer for more than 200 million years [4]. Among various cancers, prostate and breast cancer (BC) account for a significant portion of cancer cases in men and women, respectively, and brain and hematological malignancies account for the highest percentage of cancer cases among children [1,5,6,7,8]. Importantly, cancer is generated by a series of gene mutations that alter the functions of cells, and these include both oncogenes and tumor suppressor genes [1,4,9,10,11,12,13,14,15,16,17]. Several studies suggest tumorigenesis as a multistep process involving genetic changes which cause typical cells to undergo a gradual change into extremely lethal variants [18,19,20]. Interestingly, several biomarkers have been discovered to have diagnostic and therapeutic utility, and globally increasing cancer incidence and mortality bring about a need for precise biomarkers for better detection, diagnosis, prognosis, and monitoring [11,21,22,23,24]. Currently, only a few cancer biomarkers are advised for clinical use, with the majority of them being used to assess treatment response in advanced cancer patients. However, the majority of cancer biomarkers now being used in clinical settings are less effective for mass screening or early diagnosis, and only a few new and helpful cancer biomarkers are being discovered and proven for screening and early diagnosis [24]. As a result, the discovery of novel biomarkers is needed for the identification and detection of cancer.

**Figure 1:**
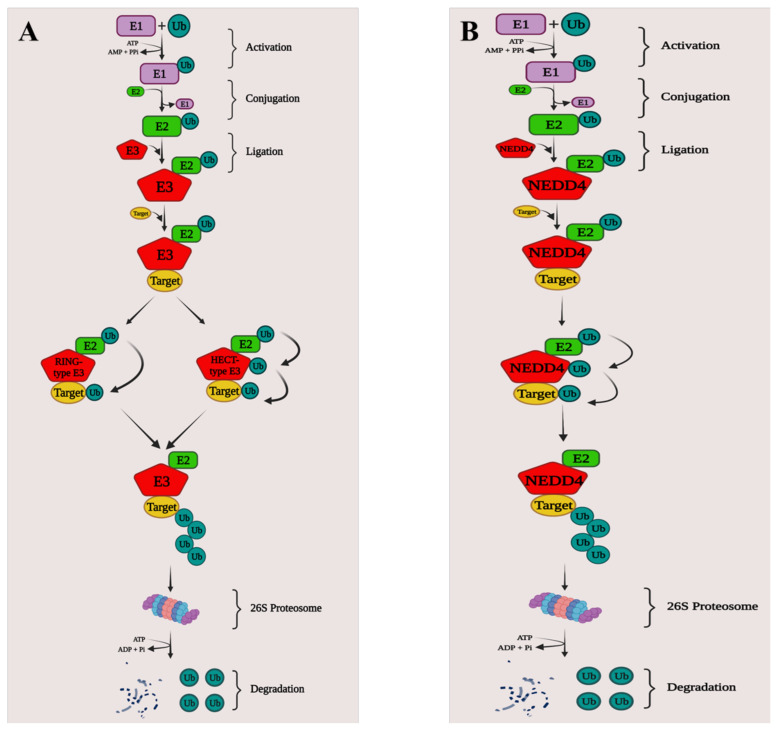
**Mechanistic role of E3 ligases ubiquitinating target substrates**. (**A**) The E1, E2, and E3 catalyze ubiquitination. E1 activates ubiquitin in an ATP-dependent manner before transferring it to E2. E2 then transfers the ubiquitin to E3 which in turn transfers to target substrates for degradation by the 26S proteasome. In the case of RING-type E3s, the transfer of ubiquitin to the target substrate requires an intermediate complex of the target, E3, E2, and Ub. For HECT-type E3s, prior to ubiquitin transfer to the target, E2 with Ub binds and transfers the ubiquitin to the E3. (**B**) NEDD4 is a HECT-type E3 ubiquitin ligase, ubiquitinated by E2, which then transfers the ubiquitin to the target substrate leading to the degradation by the proteasome. Created using BioRender.com.

The ubiquitin-proteasome system is an essential form of post-translational protein modification (PTM) that is important in protein homeostasis, as well as in the regulation of vital physiological activities [25,26,27]. Ubiquitination is extremely important in biological processes and alters post-translationally modified proteins in a way that causes the degradation, stabilization, or relocation of substrates [27,28,29,30,31,32]. The ubiquitin-mediated proteolytic pathway is a highly adaptable and reversible process mediated by an enzyme cascade [27]. Ubiquitination regulates thousands of intracellular protein levels in cells and plays a role in practically all cell physiology and disease, including DNA damage and repair, cell death, and immunological responses, by initiating selective proteolysis via the 26S proteasome [27,33,34]. Ubiquitin (Ub) is a highly conserved regulatory protein with 76 amino acids that can covalently tag target proteins through a series of enzymatic processes involving the enzymes Ub-activating (E1), Ub-conjugating (E2), and Ub-ligating (E3) [35,36,37,38]. Deubiquitinases (DUBs), which are also essential for nearly all cellular signaling pathways, including the cell cycle, apoptosis, receptor downregulation, and gene transcription, can similarly reverse the activity of Ub ligases by removing Ub from substrate proteins [39,40]. Ubiquitination is activated via a sequential cascade including E1, E2, and E3, with E1 first triggering Ub via an ATP-dependent process [35,41]. The activated Ub is then transported to an E2 catalytic cysteine residue and finally binds to the specific target (Figure 1) [42,43,44].

E3 ligases play the most important role in recognizing the target protein and regulating the covalent connection between the target and ubiquitin moieties in this enzyme cascade [44]. The human genome already has 600 E3s, which are mostly divided into three groups: E3s from the RING finger family, the RING-between-RING (RBR) family, and the E6-AP C-terminus (HECT) family [45]. The E3 subfamily’s pivotal members are HECT-type E3s, which have a typical C-terminal module containing 350 amino acids [46,47,48]. These E3s are classified into three groups, HERC (HECT and RCC-like domain) E3s, neural precursor cell-expressed developmentally downregulated 4 (NEDD4)/NEDD4-like E3s, and other E3s [46,47,48].

Numerous studies have reported that tumor formation and incidence have been linked to abnormalities in ubiquitination [40,49,50]. As E3s are known to have a significant role in the system’s substrate specificity, their abnormalities might contribute to the development of human cancer [51,52]. Moreover, ubiquitination tightly regulates components in both tumor-suppressing and tumor-promoting pathways [53,54]. There have been many targeted therapies developed to battle carcinogenesis based on altered components such as the E3 ligases, E1, E2, deubiquitinases (DUBs), and proteasomes [27,55,56]. Among those, E3 ubiquitin ligases (E3s) are crucial ubiquitination enzymes and play a vital role in cancer development [53,57]. Moreover, ubiquitin ligase mutations and changes are seen in a diverse spectrum of tumors and have a significant impact on clinical outcomes [53,58,59]. In several studies, it was reported that among the HECT family E3s, the function of NEDD4/NEDD4-like E3 ligases influences cancer cell proliferation, migration, and invasion, as well as anticancer therapy sensitivity, via regulating several substrates [53,54,60].

**Figure 2:**
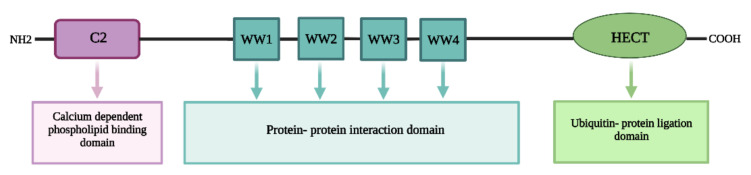
**General structure of NEDD4/NEDD4-like E3 ligase**. Contains an N-terminal C2 domain for membrane binding, a central two to four double tryptophan residue (WW) domain for protein–protein interaction and a C-terminal HECT domain for ubiquitin–protein ligation, Created using BioRender.com.

The NEDD4/NEDD4-like E3 ligase is a ubiquitin–protein ligase that could regulate a variety of membrane proteins to help them internalize and turnover [61]. It also has a significant impact on central nervous system development, hypertension regulation, etc. [45]. The NEDD4 protein is mostly found in the cytoplasm, particularly near the nucleus, and in humans the NEDD4 gene is located on chromosome 15q21.3 and has 33 exons that code for 120 kDa protein [54,62,63]. The NEDD4 structure has a C-terminal HECT domain for ubiquitin–protein ligation, an N-terminal C2 domain for membrane binding, and a central two to four double tryptophan residue (WW) domain for protein–protein interaction (Figure 2) [64]. The C2 domain is a 116-amino-acid-long calcium-dependent lipid-binding region that directs proteins to phospholipid membranes and functions in protein–protein interactions. WW domains regulate the protein–protein interactions that are typically 40 amino acids long and hold two conserved tryptophan (W) residues separated by 21 amino acids. A conserved cysteine residue in the HECT domain creates an intermediary thioester bond with active ubiquitin received from an E2 before catalyzing lysine ubiquitination in the substrate protein [64,65,66,67].

**Figure 3:**
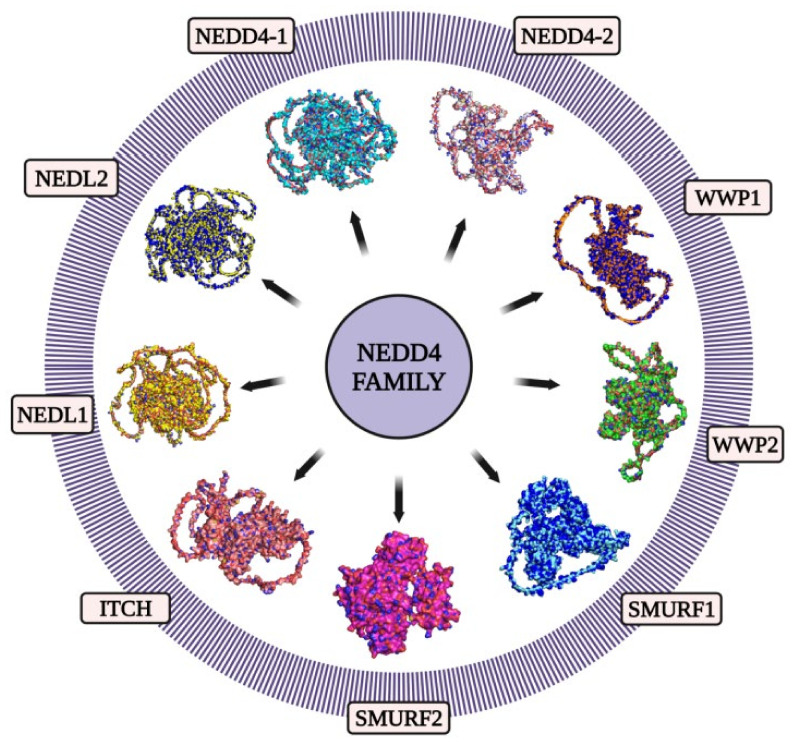
**Structural representation of the founding members of the NEDD4 family of E3 HECT ubiquitin ligases in humans:** NEDD4-1 (UniProt ID: P46934), NEDD4-2 (UniProt ID: Q96PU5), WWP1 (UniProt ID: Q9H0M0), WWP2 (UniProt ID: O00308), SMURF1 (UniProt ID: Q9HCE7), SMURF2 (UniProt ID: Q9HAU4), ITCH (UniProt ID: Q96J02), NEDL1 (UniProt ID: Q76N89), NEDL2 (UniProt ID: Q9P2P5). These proteins’ primary structures were taken from the UniProt database. Using the AlphaFold protein structure database, the structures of these proteins were predicted. The image generation and visualization of the structures of these proteins were conducted using PyMOL [88,89,90,91]. Created using BioRender.com.

**Figure 4:**
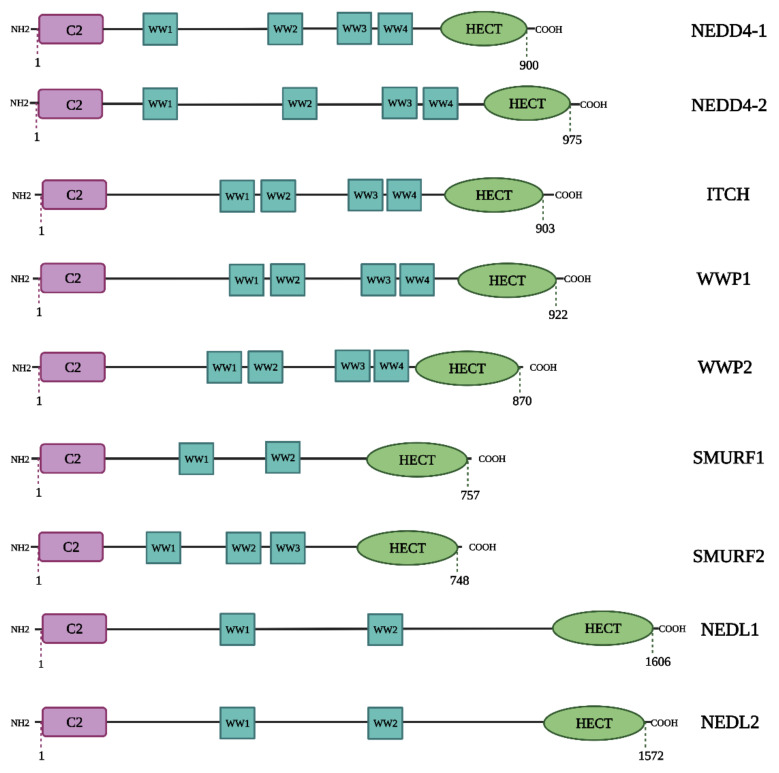
**Structures of the 9 NEDD4 subfamily proteins.** NEDD4 subfamily ligases contain a C2 domain, 2–4 WW domains that recognize PY motifs, and a HECT domain. Created using BioRender.com.

In mammals, the NEDD4 family consists of nine members: NEDD4, NEDD4 Like E3 Ubiquitin Protein Ligase (NEDD4L) also known as NEDD4-2, the SMAD Ubiquitylation Regulatory Factors 1 (SMURF1) and 2 (SMURF2), WW Domain Containing E3 Ubiquitin Protein Ligase 1 (WWP1) and 2 (WWP2), NEDD4-like ubiquitin–protein ligase 1 (NEDL1) and 2 (NEDL2) and the Itchy E3 Ubiquitin–Protein Ligase Homolog protein (ITCH) (Figure 3) [62]. All of these members of the NEDD4 subfamily share a common functional domain structure (Figure 4) [68,69]. The NEDD4 subfamily members are involved in a variety of cellular processes. The proteins NEDD4 and NEDD4-2 were the first members of this subfamily to be found and are now the most investigated. The subfamily members are considered to be involved in a variety of illnesses, including cancer and neurological diseases [60,70].

NEDD4-2 functions in a variety of ion channels, including chloride, potassium, and sodium [71,72,73]. It also interacts with proteins involved in the Wnt signaling pathway, the TGF*-β* signaling pathway, and the autophagy process [74,75,76]. ITCH regulates a wide range of biological mechanisms because of a large number of target proteins [77]. In addition, ITCH regulates the TGF-*β* signaling pathway and influences cancer [78]. Another member, WWP1, is a versatile protein that has many targets including ErbB4/HER4, JunB, Smad2, Smad4, and p53. As a result, WWP1 is involved in cancer, protein degradation, protein trafficking, transcription, viral budding, and infectious and neurological diseases [79]. WWP2 binds to substrates in many signaling pathways, including the PI3K/Akt and TGF-*β* pathways, and is linked to cancer and immune system modification [80]. Both SMURF1 and SMURF2 suppress the TGF-*β*/BMP signaling pathways [81,82]. SMURF1 is involved in the noncanonical Wnt and MAPK pathways and regulates cell growth and morphogenesis, cell migration and polarity, and autophagy [83]. SMURF2 has been linked to similar pathways. Its dual role in cancer is frequently studied, as it can serve both as a tumor suppressor and an oncoprotein [83]. NEDL1 and NEDL2 are also known as the HECT, C2, and WW domains containing E3 ubiquitin–protein ligase 1 (HECW1) and 2 (HECW2) [70]. By ubiquitinating and degrading Dishevelled-1(Dvl1), NEDL1 participates in the Wnt signaling pathway [84,85]. Recent evidence suggests that NEDL1 is also involved in the TGF-*β* signaling pathway via Smad4 ubiquitination. These two proteins, HECW1 and HECW2, appear to interfere with a variety of physiological functions, including the enteric nervous system and kidney development [86,87].

NEDD4 mediates receptor-mediated endocytosis along with proteasomal degradation and ubiquitination of its substrate proteins [63]. NEDD4 E3 ligases modulate signaling molecule trafficking by monoubiquitination and K63-linked polyubiquitination and hence play a critical role in cellular activities [63]. The NEDD4 is an important regulator of various cellular physiological processes such as endocytosis, cell growth, neuromuscular junctions, and modulation of epithelial Na+ channels (ENaC) [92,93,94].

In addition, NEDD4 is also linked to several diseases, including cancer, cardiovascular disease, metabolic disease, neurological disorders, renal disease, and others. For example, reduced NEDD4 function causes significant heart arrhythmia by changing cardiac ion-channels following transcription [95]. Further, adult mice with conditional NEDD4L deletion in lung epithelial cells were shown to develop chronic lung illness with increasing fibrosis and bronchiolization. These mice also showed increased Muc5b expression in peripheral airways, honeycombing, and distinctive changes in the lung proteome [96]. Another in vivo study found that NEDD4-2 loss causes an unanticipated progressive kidney damage phenotype that includes increased Na^+^ reabsorption, increased Na^+^Cl^−^ and ENaC cotransporter expression, hypertension, and low aldosterone levels in animal models [71].

## 2. NEDD4 and Cancer

NEDD4 has a significant role in the development and progression of various types of cancers by targeting different substrates. Interestingly, it is found that there are many genes upstream and downstream of NEDD4, and it has the potential to be employed as a molecular switch to control tumor development via these competitive substrates [54]. Expectedly, NEDD4 has been associated with the regulation of several signaling pathways. For example, the WW domain of NEDD4 contributes to polyubiquitination and degradation of Smad2/3, limiting TGF-*β* signaling by specifically recognizing the TGF-*β*-induced phospho Thr-ProTyr motif in the junction region [74]. Further, NEDD4 causes the ubiquitination of Unc-51 like autophagy activating kinase 1 (ULK1), a protein involved in the initiation of autophagy, and controls the process [75].

According to several studies, NEDD4 acts as an oncoprotein that promotes cancer cell development. For example, a study demonstrated that siRNA reduction of NEDD4 resulted in a decrease in cell proliferation and changed cell shape in LoVo and HCT-15 colon cancer cells. According to Amodio et al., NEDD4 overexpression was found in non-small-cell lung cancer (NSCLC) cell lines, while NEDD4 knockdown in vitro and in vivo dramatically decreased NSCLC cell proliferation and tumor growth, respectively. NEDD4’s carcinogenic effect in NSCLC cells can be due to the inactivation of PTEN [97]. NEDD4 is the key pro-oncogenic ligase that mediates the ubiquitination of PTEN; however, NEDD4L is mainly involved in the degradation of the tumor suppressor gene p53 [98,99,100]. Moreover, in breast and prostate cancer cells, NEDD4 might enhance cell proliferation and migration by regulating the PTEN/Akt signaling pathway [101]. However, Eide et al. reported that NEDD4 increased colon cancer cell proliferation without relying on the PTEN or PI3K/Akt signaling pathways [102]. A study reported that NEDD4 targets Myc oncoproteins, c-Myc and N-Myc, for degradation by directly binding to them, which resulted in downregulation of these proteins and suppression of cell proliferation in pancreatic cancer and neuroblastoma [63,103]. Several NEDD4 substrates, such as Ras, Notch, pAkt, VEGFR2, Mdm2, and FGFR, have been identified as oncoproteins in carcinogenesis, suggesting that using NEDD4 to target their breakdown could have anticancer effects [63].

A plethora of studies have shown that NEDD4 promotes tumor growth in diverse types of cancers, and these studies suggest that the role of NEDD4 in tumor progression is context-dependent [60,63]. This pleiotropic nature of NEDD4 is mainly attributed to its ubiquitous expression in a wide range of tissues. NEDD4 proteins have several downstream target substrates that arbitrate various functionalities, and numerous receptors that share the same protein trafficking and endocytic machinery are controlled by various NEDD4-like E3s [60]. Therefore, in malignancies, NEDD4 behaves as both an oncogene and a tumor suppressor, thus NEDD4 inhibitors or activators are needed for cancer treatments [54,63]. NEDD4 was found to be an oncogene because of its role in inhibiting PTEN, a well-known tumor suppressor [104]. In several types of human cancer cell lines, PTEN degradation and increased NEDD4 levels have been discovered [104]. PTEN is a phosphatase that inhibits tumor growth in a variety of malignancies by suppressing the PI3K/Akt signaling pathway, which is required for the survival of cancer cells. NEDD4 has been identified as a PTEN-specific E3 ligase and has been shown to promote Akt signaling by lowering the amount of PTEN protein. Thus, NEDD4 has been proposed as an oncoprotein and a possible drug target [60]. Aforementioned studies highlight NEDD4 as a potential biomarker for poor prognosis as well as a therapeutic target for the treatment of different cancer types.

**Figure 5:**
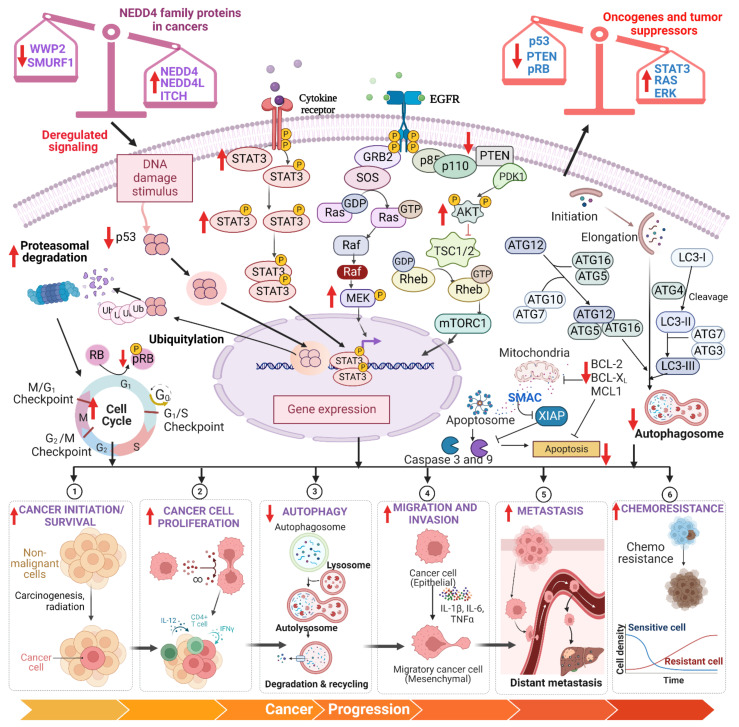
**Role of NEDD4 family proteins in cancer development and progression.** A plethora of studies have shown that NEDD4 family proteins are deregulated in various cancers. The imbalance in the expression of tumor-growth-promoting NEDD4 family proteins such as NEDD4, NEDD4L, ITCH, and WWP1 and tumor-growth-suppressing NEDD4 family proteins such as WWP2 and SMURF1 independently leads to increased proteasomal degradation of p53, downregulation of PTEN and pRB, and upregulation of STAT3, Akt, MEK, MMP-1, and MMP-13. This disproportion in the expression of tumor suppressors and oncogenes promotes cancer cell initiation, survival, proliferation, migration, invasion, metastasis, and chemoresistance and inhibits apoptosis and autophagy. Created using BioRender.com.

In the current article, we discuss the function of the NEDD4 protein in the development and suppression of cancer. Then, we describe the vital roles of NEDD4 in carcinogenesis and development, which include cell survival, cell proliferation, autophagy, cell migration, invasion, metastasis, epithelial–mesenchymal transition (EMT), chemoresistance, multiple signaling pathways, oncogenic role, and tumor-suppressive role (Figure 5). These studies suggest that NEDD4 can be used as a drug target for human cancer; therefore, we briefly discuss the clinical significance of NEDD4 in this article.

## 3. NEDD4 Functions in Human Cancers

### 3.1. NEDD4 and Cell Survival

NEDD4 is known to induce cancer cell survival in different cancers. For example, NEDD4 overexpression leads to the suppression of PTEN expression and induces Notch-1, which leads to decreased apoptosis in RT4 bladder cancer (BCa) cells. Moreover, this study also showed that suppression of NEDD4 leads to BCa cell apoptosis [105]. Similarly, in QGY7703 and SMMC7721 liver cancer cells, NEDD4 acts as an oncoprotein, and overexpression of this protein was shown to suppress apoptosis via partially increasing large tumor suppressor kinase 1 (LATS1) expression [106]. Moreover, LATS1/2 is ubiquitinated by NEDD4 when it recognizes the PY motif in proteins [107]. In addition, NEDD4 downregulation resulted in DU145 cell apoptosis via both death receptor and mitochondria-dependent mechanisms [108]. A recent study showed that overexpression of FOXA1 reduces the proliferation induced by NEDD4 upregulation and promotes apoptosis in Caco-2/LoVo colon cancer cells [109]. In conclusion, NEDD4 has a crucial role in the survival of cancer cells.

### 3.2. NEDD4 and Cell Proliferation

The function of NEDD4 in cancer cell proliferation is demonstrated by several studies. For example, the overexpression of NEDD4 increased the proliferation in HeLa cells [108]. Similarly, in QGY7703 and SMMC7721 liver cancer cells, the overexpression of NEDD4 suppressed LATS1, which results in enhanced cell proliferation [106]. Further, NEDD4 knockdown inhibits cell proliferation in Huh-7 HCC cells through overexpression of PTEN and consequent inactivation of Akt, ERK1/2, and STAT3 [110]. A similar study also revealed that in Huh7 HCC cells, depletion of NEDD4 has a detrimental effect on cell proliferation. NEDD4 appears to be important in promoting HCC proliferation and spread through the stimulation of the PTEN/PI3K/Akt signaling pathway [111]. In PANC-1 pancreatic ductal adenocarcinoma (PDAC) cells, NEDD4 depletion suppresses cell growth partially by regulating the PTEN/Akt signaling pathway [112]. In addition, downregulation of NEDD4 suppresses proliferation of RT4 BCa cells. Inhibition of NEDD4 activated PTEN while suppressing Notch-1, indicating that NEDD4’s oncogenic activity was mediated partially by downregulation of PTEN and overexpression of Notch-1 [105]. Interestingly, another study suggested that NEDD4 can inhibit phosphatidylinositol 4-phosphate 5-kinase *α* (PIP5K*α*)-dependent phosphatidylinositol 4,5-bisphosphate (PIP2), leading to a significant proliferation defect [113]. In conclusion, these studies have established that NEDD4 can affect proliferation in various cancer cells.

### 3.3. NEDD4 and Autophagy

Autophagy is an evolutionarily conserved catabolic mechanism that eukaryotic cells use to degrade or recycle internal contents through a membrane-trafficking pathway [114,115]. In distinct phases of cancer development, autophagy performs a dynamic tumor-promoting or tumor-suppressing role [116,117]. It has shown that autophagy was suppressed in lung and prostate carcinoma cell lines when NEDD4 was knocked out and this NEDD4 downregulation significantly increased activated mTOR (p-mTOR) levels, implying that mTOR signaling was involved in NEDD4-mediated autophagy [108]. Another study has shown that high levels of advanced glycation end product (AGE) levels increased NF-κB expression in Hep3B and Saos-2 cell lines, which is amplified more in the absence of p53. The NF-κB-dependent expression of NEDD4 enhances Beclin 1 cleavage, impairing autophagy and causing autophagosome build-up, which leads to cell death [118]. Beclin 1 is a subunit of the PI3K-III complex and a tumor suppressor that plays an important function in autophagy and apoptosis. This protein has a sequence that looks like a PY motif (LPxY), and the WW domains of NEDD4 interact with it specifically. NEDD4 was shown to polyubiquitinate Beclin 1 and regulate its stability, which suppresses autophagy [60,119]. Further, in pancreatic cancer cells, NEDD4 prevents autophagy activity under metabolic stress by decreasing mitochondrial function and limiting tumor development by destabilizing an autophagy protein, ULK1, and a glutamine transporter, ASCT2 [75]. Taken together, NEDD4 seems to prevent autophagy in cancer cells.

### 3.4. NEDD4 and Cell Migration and Invasion

According to several studies, it was noted that NEDD4 overexpression increases cell migration and invasion in various cancers. For example, a study demonstrated that NEDD4 overexpression decreased PTEN and increased Notch-1 levels, resulting in invasion and migration of RT4 BCa cells [105]. Another study in colon cancer tissues demonstrated that NEDD4 promotes the progression of cancer by activating FOXA1 ubiquitination in conjunction with the inhibition of miRNA-340-5p and the overexpression of ATF1 [109]. Similarly, downregulation of NEDD4 using curcumin in pancreatic cancer cell lines Patu8988 and Panc-1 resulted in a considerable reduction in tumor cell development, in association with increased expression of PTEN and p73 [120]. Further, in QGY7703 and SMMC7721 hepatocellular carcinoma (HCC) cells, NEDD4 increased cell migration and invasion via inhibiting the tumor suppressor LATS1 [106]. Another study reported that NEDD4 is closely linked to the development and progression of lung cancer due to its overexpression in a significant fraction of NSCLC, promotion of PTEN degradation, and enhancement of the malignant characteristics of lung epithelial cells [97]. Similarly, an in vivo study in NSCLC showed that silencing of NEDD4 inhibited cell invasion and migration by increasing PTEN expression and by inhibiting PI3K/Akt signaling pathway [121]. Moreover, NEDD4 knockdown in A549 NSCLC cells prevented EGF-induced cell migration through regulating its interaction with the EGFR signaling complex and cathepsin B lysosomal secretion [122,123]. According to another study, the influence of proline-rich *γ*-carboxyglutamic acid protein 4 (PRRG4) on invasion and migration in BC cells is mediated via both NEDD4 binding and Robo1 downregulation [124]. In U251 glioma cells, it was reported that NEDD4 overexpression enhanced the cell invasion and migration due to ubiquitination and degradation of cyclic nucleotide ras guanine nucleotide exchange factor (CNrasGEF) [60]. Moreover, NEDD4 promoted the ubiquitination of NEDD4/Rap2a, which eased the invasion and migration of U251 and U87 cancer cells. In addition, human glioma cells showed suppression of invasion and migration when NEDD4 was downregulated, but overexpression of NEDD4 reversed these effects. These findings imply that NEDD4 has a significant role in glioma development [125]. It was also shown that NEDD4 mediates EGF-dependent AGS and N87 gastric cancer cell line invasion and migration via EGFR-mediated metastasis signaling [126]. Taken together, NEDD4 increases cell migration and invasion in human cancer cells.

### 3.5. NEDD4 and Metastasis

Metastasis is the process by which cancer cells spread from the primary tumor to surrounding tissues and distant organs, and it is the leading cause of cancer morbidity and mortality [127,128,129]. In several studies, NEDD4 has been described to take part in the progression of metastasis. For example, the stability of NEDD4 is regulated by p34^SEI-1^, a 34 kDa oncoprotein that controls PTEN degradation and induces the PI3K/Akt pathway, which results in tumorigenesis and metastasis [130]. Another study revealed that PRRG4-mediated recruitment of NEDD4 stimulates ubiquitination and degradation of Robo1 (a tumor suppressor gene), subsequently leading to the activation of protein tyrosine kinases Src and FAK, which are critical for BC cell motility, invasion, and metastasis [124]. An in vivo study demonstrated that overexpression of NEDD4 in CC tissues and Caco-2/LoVo cell lines aided in the development of xenograft tumors and metastasis, as well as tumorigenesis in mice models [109]. Interestingly, another study showed NEDD4 to be important in gastric cardia adenocarcinoma (GCA) metastasis, as the shRNA-mediated NEDD4 decrease in AGS and N87 gastric cancer cells impairs basal and EGF-stimulated cell invasion and migration [126]. It was also shown that NEDD4 is important for lung cancer metastasis by increasing cathepsin B secretion or causing PTEN degradation. However, NEDD4-2 has more complex functions than NEDD4 as it inhibits lung cancer cell metastasis; on the other hand, it enhances lung cancer survival by causing general control nonderepressible 2 (GCN2) degradation [123]. Further, NEDD4 appears to play a significant role in promoting HCC metastasis via stimulating the PTEN/PI3K/Akt signaling pathway [111].

### 3.6. NEDD4 and EMT

The epithelial–mesenchymal transition (EMT) is a process that can be reversed and temporarily transforms epithelial cells into quasi-mesenchymal cell types, and this process plays a significant role in chemoresistance and metastasis [131,132,133,134,135,136]. In CNE1 and CNE2 nasopharyngeal carcinoma cells, it was found that partial overexpression of the NEDD4 signaling pathway caused EMT in DDP-resistant cells. Moreover, EMT shifts to MET when NEDD4 is deleted via an shRNA, making DDP-resistant cells susceptible to DDP [137]. NEDD4 has been linked to lysosomal-associated protein transmembrane 5 (LAPTM5), and the downregulation of LAPTM5 in T24 and 5637 BCa cells suppressed proliferation and hindered invasion and migration through the inhibition of EMT markers, which hinted that NEDD4 can be involved in LAPTM5-influenced EMT [138]. Another study showed that miR-93 overexpression in Huh-7 cancer cells facilitated TGF-*β*-induced EMT by inhibiting NEDD4L expression [139]. Notably, NEDD4 overexpression was observed to induce the EMT, which might be responsible for its role in invasion and metastases.

### 3.7. NEDD4 and Chemoresistance

Chemoresistance is often cited as a primary cause of cancer therapeutic failure, resulting in cancer relapse and spread [140,141,142]. NEDD4 has shown to play a major role in chemoresistance in various cancers. A study on lung cancer proved that upregulation of NEDD4 via the PI3K/Akt pathway might contribute to tumor growth and modulate lung ADC chemoresistance [121]. As previously mentioned, NEDD4 is carcinogenic in CNE1 and CNE2 NPC cells because it increases the EMT features of DDP-resistant cells. It was also discovered that knocking down NEDD4 with shRNA reversed EMT and sensitized DDP-resistant cells. These data suggest that NEDD4 is involved in chemoresistance in NPC cancer cells [137]. Moreover, a study on U87MG and U251 GBM cell lines demonstrated the crucial role of NEDD4 in controlling the redox imbalance in temozolomide (TMZ)-resistant GBM cells by PTEN degradation and activation of the Akt/NRF2/HO-1 signaling pathway [143]. Another similar study in GBM revealed that glioma-linked oncogenic lncRNA LINC01198 promotes proliferation of gliomas and temozolomide resistance by acting as a scaffold and enlisting NEDD4 enzymes to attack certain proteins such as PTEN [144]. In A375 melanoma cell lines, erastin, a ferroptosis activator, stimulates NEDD4 and FOXM1 expression, and it led to the ubiquitination and degradation of voltage-dependent anion channels, VDAC2/3, which further led to the suppression of erastin-induced ferroptosis in melanoma [145]. Another study in H1650/ER cells showed that NEDD4 might enhance NSCLC-acquired resistance to erlotinib by lowering the expression of PTEN [146].

### 3.8. NEDD4 and Multiple Signaling Pathways

NEDD4 is a protein that regulates many signaling pathways. Wnt/*β*-catenin signaling is negatively regulated by NEDD4, which targets the LGR5 receptor and DVL2 for proteasomal and lysosomal degradation. Moreover, inactivation of NEDD4 and NEDD4L promotes Wnt activation, which improves tumor propensity and progression [147]. On the other hand, knockdown of NEDD4 decreases pAkt levels, increases PTEN, and inhibits the development and migration of the Hep3B HCC cell line [60,111]. According to another study, NEDD4 promotes insulin-like growth factor-1 (IGF-1) signaling and mitogenic activity by interacting and conjugating monoubiquitin to IRS-2, improving IRS-2-mediated signaling and cell proliferation triggered by IGF-1. Interestingly, in PC-3 prostate cancer cells, the NEDD4 and IRS-2 connection is also essential for optimal IGF-1 signaling activation and cell proliferation [148]. Another study showed that NEDD4 ubiquitylates and destabilizes WW45 kinase and LATS1/2, which are needed for active Hippo signaling. NEDD4 positively regulates cell proliferation through the negative regulation of LATS1/2 and WW45. This suggests that NEDD4 influences cell proliferation and cancer growth through Hippo signaling. As a result, NEDD4 functions as a regulator in the Hippo pathway, and the depletion of NEDD4 causes an increase in apoptosis and a decrease in proliferation [149].

## 4. Effect of NEED4 on Various Types of Cancer

A surfeit of studies suggests that the NEDD4 protein has both oncogenic and tumor-suppressive properties. Several preclinical studies revealed that NEDD4 has cancer chemopreventive and therapeutic capabilities against various malignancies, which include solid tumors such as brain, breast, colon, bladder, liver, pancreas, prostate cancers, etc., and hematological cancers such as lymphoma and leukemia. Table 1 and Table 2 summarize the role of the NEDD4 protein in various types of cancers. These studies, which are briefly discussed below, reveal that the NEDD4 protein has immense potential as a successful target for both the prevention and treatment of many malignancies.

### 4.1. Bladder Cancer (BCa)

Bladder cancer (BCa) is one of the most frequently occurring malignancies of the urinary bladder, which accounts for 3 percentage of global cancer diagnoses according to GLOBOCAN 2020 [105,138,200,201,202]. Interestingly, it was noted that downregulation of NEDD4 resulted in the inhibition of cell proliferation, invasion, and migration in RT4 BCa cells. Moreover, NEDD4 inhibition activated PTEN while suppressing Notch-1. Therefore, downregulation of NEDD4 in BCa cells reduced cell proliferation, induced apoptosis, and hindered cell migration [105]. This study pointed out the importance of the development of NEDD4 inhibitors for the treatment of human BCa.

### 4.2. Breast Cancer (BC)

BC is the most frequent type of cancer in women all over the world, and triple-negative breast cancer (TNBC) is characterized as a very aggressive malignancy with a bad prognosis and no viable targeted therapy available for its treatment [203,204,205,206,207,208,209,210]. NEDD4’s role in the development of BC has been reported in several studies [54,104,178,211]. It has been observed that NEDD4 is predominantly overexpressed in HER2-amplified BC [212]. The expression of NEDD4L was also reported to be associated with a better prognosis for BC survivors [213]. A study revealed p34^SEI-1^ as a highly expressed oncoprotein in human BC tissues which causes tumorigenesis by triggering NEDD4 which in turn causes poly-ubiquitination and degradation of PTEN, thereby promoting tumorigenesis by favorably regulating the PI3K/Akt pathway [151]. According to another study, PRRG4’s influence on migration and invasion in BC cells is mediated via both NEDD4 binding and Robo1 downregulation [124]. Another in vitro study suggests that using the MDA-MB-231 cell line, Cdh1 suppressed the E3 ligase activity of WWP2 in the MDA-MB-231 cell line in a manner that is distinct from the anaphase-promoting complex and cyclosome. Therefore, the lack of Cdh1 activates WWP2, which in turn causes a decrease in the abundance of WWP2 substrates such as PTEN, which stimulates PI3K/Akt oncogenic signaling and promotes cancer growth [173]. Another study in the MDA-MB-231 cell line revealed that the expression of the tumor suppressor Mig6 is stabilized by type I *γ* phosphatidylinositol phosphate 5-kinase i5 (PIPKI*γ*i5). Mig6 expression is lost as a result of PIPKI*γ*i5 knockdown, which significantly increases and prolongs EGFR-mediated cell signaling. Direct interaction between PIPKI*γ*i5 and NEDD4 disrupts the process of Mig6 ubiquitination and subsequent proteasomal destruction. Therefore, in PIPKI*γ*i5-knockdown cells, a lack of NEDD4 can restore the expression of Mig6 [174]. NEDD4 was more likely to bind to connexin 43 (Cx43) when ER was inhibited, which caused Cx43 to be ubiquitinated. Additionally, tamoxifen and fulvestrant suppressed ER and phosphorylated the MAPK subunit p38, and downregulated Rac and MKK3/6. Further, pretreatment with Akt and MAPK inhibitors reversed fulvestrant-reduced Cx43 expression. These results demonstrate that in ER-dependent ER-positive BC cells and Cx43 expression might positively influence cell motility [177]. ARRDC3 (arrestin domain-containing 3) is a metastasis suppressor that blocks EGF-driven endocytic recycling of ITG*β*4 by promoting NEDD4-dependent ubiquitination. By lowering ITG*β*4 levels in EVs, ARRDC3 lowers the metastatic capacity of BC-cell-derived extracellular vesicles (EVs) [175]. Histopathological evaluation has shown that WWP1, as well as NEDD4 expression, were modulated in the BC tissues [150,152]. Moreover, knockdown of WWP1 in MCF-7 and T47D BC cells has shown a decrease in cell growth and colony formation. Hence, WWP1 might function as a co-activator or anti-apoptotic factor to encourage the survival and growth of BC cells [150]. Another study in MCF-7 and T47D cell lines reported that WWP1 negatively regulates LATS1, a tumor suppressor, through its degradation by polyubiquitination and the 26S proteasome pathway. Moreover, it was reported that the knockdown of NEDD4 and ITCH increased the level of LATS1, whereas the knockdown of SMURF1 decreased the level of LATS1 [171]. In addition, robustaflavone-A (RF-A) dramatically increased MCF-7 ferroptosis, resulting in lipid peroxidation and ROS generation by increasing the expression of VDAC2 channels and decreasing the expression of NEDD4 [180]. Apart from the oncogenic property of NEDD4 revealed in several studies, some reports state the tumor-suppressing role of NEDD4 in BC. For example, a study has demonstrated the tumor-suppressing role of NEDD4 by which PIP5K*α*, a ubiquitinated protein that enhances the proliferation, invasion, and migration of BC cells, was impeded by NEDD4. PIP5K*α* produces PIP2, a key regulator of lipids in a variety of physiological activities. The oncogenic PI3K/Akt pathway can also act upstream via PIP5K-dependent PIP2 production. PIP5Ka was ubiquitinated and proteasomally degraded by NEDD4, resulting in a decrease in plasma membrane PIP2. Furthermore, deletion of the PIP5Ka gene reduced EGF-induced Akt activation and produced a major proliferation deficit [113]. In another study, the knockdown of NEDD4 showed to increase the levels of MAPK phosphatase 3 (MKP3) in MDA-MB-231 BC cells [170]. Another study demonstrated a negative correlation between HER3, a key player in cancer, and NEDD4 levels. More significantly, the NEDD4 knockdown increased HER3 expression, sensitizing cancer cells to the anti-HER3 antibody’s ability to suppress cell proliferation. Collectively, these findings imply that low NEDD4 levels might be able to anticipate when HER3 signaling is activated. In addition, NEDD4 knockdown along with NRG-1 and HER3 mAb in an MCF-7 (shNEDD4) mice xenograft showed decreased tumor volume [172]. Further, an in vitro study showed decreased ER and HER3 expression and increased proliferation rate in shNEDD4 knockdown MCF-7 cells [176]. Another in vitro study demonstrated that SMURF1 knockdown increased HER2 expression in BT474 cells [179]. In summary, NEDD4-like E3 ligases were found to be a feasible therapeutic target as well as a potential prognostic predictor for BC.

### 4.3. Cervical Cancer

Cervical cancer is the fourth most prevalent cancer in women worldwide and it is the most common gynecological malignancy in developing nations, and has an incidence rate of less than 1 percentage and mortality of less than 0.5 percentage [214,215,216,217,218,219]. A study in HeLa cells reported that NEDD4 prevented PTEN from inducing apoptotic cell death, and PTEN, in turn, decreased the level of NEDD4 [181]. Another study in HeLa cells described NEDD4 as a novel binding partner of Beclin 1, and this study showed that NEDD4 polyubiquitinates Beclin 1 with Lys11 and Lys63 linked chains. Importantly, NEDD4 expression regulates Beclin 1 stability, and it is degraded by the proteasome via Lys11-linked polyubiquitin chains when the Beclin 1 interacting protein VPS34 is depleted. Therefore, Beclin 1 is the first tumor suppressor to be described as being under the control of Lys11-linked polyubiquitination [119]. Further, NEDD4L overexpression specifically downregulates ULK1 protein levels by preferentially ubiquitylating it for degradation by the proteasome, which actively transcribes ULK1 mRNA. Basal levels of ULK1 are immediately restored upon reactivation of mTOR-dependent protein synthesis; however, mTOR inhibits the function of newly synthesized ULK1. This gets the cell ready for another round of potential autophagy stimulation in HeLa cells [182]. Furthermore, a study in HeLa cells demonstrated that NEDD4 controls the level of Cx43, which acts as a tumor suppressor both at basal conditions and in response to protein kinase C activation. Additionally, it was discovered that NEDD4 expression led to the total loss of gap junctions and an increase in the lysosomal degradation of Cx43 in HeLa cancer cells [93].

### 4.4. Colorectal Cancer (CRC)

Colorectal cancer is one of the most common cancers in women and men worldwide. Both hereditary and environmental factors play a key role in colorectal cancer etiology [220,221,222]. A study revealed that NEDD4 was found to be expressed in 80 percentage of colorectal carcinomas and has tumorigenic activity by inducing ubiquitination and degradation of the PTEN gene [153]. Another study on HCT-15 and LoVo colon cancer cells observed that PTEN and PI3K/Akt signaling are not required for NEDD4 to induce colon cancer cell growth. Moreover, NEDD4 knockdown induced alterations in cell morphology and reorganization of the actin cytoskeleton [102]. Another study demonstrated that the expression of NEDD4 has a proportional effect on cell growth and metastasis by varying vimentin, N-cadherin, snail, and ATF-1 expression in LoVo and Caco-2 cells. Further, in LoVo cells, the overexpression of NEDD4 reduced the expression of E-cadherin, FOXA1, and miR-340. Furthermore, in Caco-2 cells, overexpression of NEDD4 increased cell proliferation and colony number and reduced apoptosis by decreasing Cyto-C, PUMA, Apaf-1, Bax, and FOXA1 expression [109]. However, a couple of studies demonstrated the tumor-suppressive role of NEDD4 in colon cancer. For example, a clinical study discovered that NEDD4 is upregulated and NEDD4L downregulated in CRC tissues. Interestingly, patients with high expression of NEDD4L have shown to have longer disease-specific survival compared to patients with lower expression [76]. In CRC, NDRG1 prevented tumor growth by increasing p21 expression and decreasing its ubiquitylation. NDRG1 was found to be downregulated in CRC tissues, and a positive connection between NDRG1 and p21 was shown in vitro and in vivo. Mechanistically, NEDD4 directly interacts with p21 and targets it for degradation, which is inhibited by NDRG1, resulting in reduced growth of tumors. Further, it was demonstrated that the knockdown of NEDD4 with siRNA increased the expression of both p21 and NDRG1 [183]. Taken together, more studies are required for the proper understanding of the function of NEDD4 in CRC.

### 4.5. Endometrial Cancer (EC)

EC is the most frequent gynecologic malignancy in women, with an anticipated 61,880 new cases and 12,160 deaths in 2019 [223,224,225]. A study demonstrated the relationship between NEDD4 and EC, in which the cancer tissues showed increased levels of NEDD4. Moreover, immunohistochemistry analysis revealed strong NEDD4 staining, with higher NEDD4 expression in the most aggressive tumors. Furthermore, NEDD4 expression in EC was found to be favorably linked with the Akt downstream effector FoxM1 and increased cell growth, p-ERK, pAkt, and IGF-1R expression [155]. On the contrary, a clinical study showed considerably lower levels of NEDD4L expression in EC patients than those with benign endometrial diseases [154]. However, there is a paucity of evidence on the association between NEDD4 levels and the onset, progression, and prognosis of EC.

### 4.6. Gastric Cancer (GC)

Gastric carcinoma (GC) is one of the most common and deadly cancers in the world [226,227,228]. A study revealed that NEDD4 was found to be expressed in GC and has tumorigenic activity by inducing ubiquitination and degradation of the PTEN [153]. To date, it has been reported that NEDD4 is overexpressed in 83 percentage of GC tumors. NEDD4 is probably involved in the signaling that leads to tumor metastasis caused by the EGFR because NEDD4 facilitates EGF-dependent GC cell invasion and migration [126]. Further, a high level of NEDD4L was associated with a low level of HIF-1*α*, indicating a positive prognosis [158]. In addition, another study revealed tumors that exhibited negative NEDD4L expression were highly linked to GC invasion and metastasis [157]. Another study demonstrated the prognostic value of this protein in GC. Decitabine (DAC), an inhibitor of DNA methylation, was found to increase NEDD4 expression in MGC803 GC cells, thus promoting the cells’ ability to invade and migrate [184]. However, to determine the role of NEDD4 in the development of GC, more in vitro, in vivo, and clinical research are needed.

### 4.7. Glioma/Glioblastoma

In adults, glioblastoma is the most common form of adult brain cancer and is one of the top ten malignant tumors, despite surgery, radiation therapy, and chemotherapy, with an average nine-month survival time [161,229,230]. NEDD4 was shown to promote cell motility and invasion of malignant U251 glioma cells in vitro by ubiquitinating CNrasGEF [160]. Another study in U251 and U87 demonstrated that NEDD4 regulates glioma cell motility and invasion via the NEDD4/Rap2a pathway [161]. Moreover, it was shown that FOXM1 overexpression upregulated NEDD4, and the subsequent degradation of PTEN and activation of the Akt pathway in turn resulted in astrocyte transformation and GBM development [185]. Another study revealed that curcumin can reduce the expression of NEDD4, pAkt, and Notch1, which leads to the inhibition of cell growth and apoptosis and reduction in invasion and migration of glioma cells. Furthermore, deletion of NEDD4 sensitized glioma cells to curcumin [125]. Moreover, it was demonstrated that SMURF1 promotes the glioma cell migration and invasion and the expression of vimentin and MDM2, and the suppression of SMURF1 by siRNA transfection can reduce cell invasion and increase the p53, cleaved caspase-3, cleaved PARP, and E-cadherin [162]. Another study showed that miR-513a-5p repressed NEDD4L expression and participated in IGF-1 mediated activation of Wnt/*β*-catenin signaling, and consequently reduced TMZ cytotoxicity [186]. In human gliomas, the expression of NEDD4L is reduced, and patients with glioma who have low NEDD4L expression have a worse prognosis [159].

### 4.8. Liver Cancer

Liver cancer is an aggressive tumor that often develops from cirrhosis and chronic liver diseases [231,232]. Hepatocellular carcinoma (HCC), a primary liver cancer, is the third greatest cause of cancer-related death globally [233,234,235]. HCC, the most common type of liver cancer, is formed from hepatocytes and accounts for more than 80 percentage of all occurrences of liver cancer [236,237,238]. In HCC, the inhibition of cell proliferation, migration, and invasion, as well as cell cycle arrest in the S phase, was achieved by targeting NEDD4. Moreover, NEDD4 silencing resulted in an increase in PTEN expression, which resulted in decreased cell proliferation, migration, invasion, p-STAT3, p-Akt, and p-ERK1/2 [110]. In addition, it was observed that knockdown of NEDD4 decreased cell proliferation, viability, invasion, migration, and p-Akt levels and induced apoptosis and LATS1 expression in HCC [106]. In another study, it was reported that depletion or inhibition of NEDD4 could be used as a strategy to minimize metastasis and postpone tumor recurrence, hence improving survival rates. Moreover, NEDD4 knockdown in liver cancer cells increased PTEN and E-cadherin and decreased vimentin and p-Akt [111]. However, a study showed that NEDD4L suppressed cell growth by phosphorylating ERK1/2 and inducing apoptosis, suggesting that it might play a tumor-suppressive role in HCC by triggering MAPK/ERK-mediated apoptosis. Further, an in vivo study demonstrated that overexpression of NEDD4L prevented the growth of xenograft tumors in mice [163]. In another study, NEDD4 overexpression in HepG2 cells showed decreased guanylyl cyclase domain containing 1 (GUCD1) levels. Additionally, NEDD4 seems to influence GUCD1 degradation via the ubiquitin–proteasome system [187]. Therefore, further studies are required to confine the precise role of NEDD4 and to develop agonists/antagonists for the potential inhibition of HCC.

### 4.9. Lung Cancer

Lung cancer is the world’s top cause of death due to cancer in both men and women. It is a common cancer that is extremely aggressive and spreads quickly [14,239,240,241,242,243,244]. NSCLC accounts for 85 percentage of all lung cancers, with lung adenocarcinoma (ADC) accounting for 60 percentage of NSCLC, making ADC the most common histologic type [121,245,246,247]. It was reported that NEDD4 is linked with the progression of lung cancer and is overexpressed in a considerable proportion of NSCLC patients concerning chemosensitivity and prognosis. Thus, it promotes cell proliferation, migration, and invasion through PTEN degradation and increases the malignant characteristics of lung epithelial cells. Through stimulation of the lysosomal cathepsin B secretion pathway, NEDD4 can also mediate EGFR cell migration signaling in lung cancer cell lines [97,121,122]. A study in HCC827/ER cells demonstrated that NEDD4 might increase the acquired resistance of NSCLC cells to erlotinib by reducing the expression of PTEN. Moreover, knockdown of NEDD4 decreased tumor growth and tumor weight in nude mice (HCC827/ER cells) xenografts [146]. Another in vitro study showed that NEDD4L can act as a tumor suppressor, and it is downregulated in NSCLCs. Moreover, it was noticed that the knockdown of NEDD4L increased cell proliferation, invasion, and migration. Additionally, an in vivo study showed that knockdown of NEDD4L resulted in increased tumor growth and metastasis in nude mice (HCC827 LUC) [164]. Furthermore, another study demonstrated miR-93’s oncogenic role in lung cancer by downregulating the expression of NEDD4L. Reduced NEDD4L prevented SMAD2/SMAD3 from degrading, enhanced the TGF-*β* signal transduction, and promoted TGF-*β*-induced EMT [139]. Another study demonstrated that transmembrane prostate androgen-induced protein (TMEPAI) interacts with NEDD4 and binds to the TGF*β*-type I receptor (T*β*RI), thereby facilitating its degradation. This study also showed that NEDD4 is necessary for the transport of TMEPAI to the lysosome [188]. NEDD4 is a particular E3 ligase for GCN2, which is activated in A549 cancer cells to increase tumor aggressiveness and survival for ubiquitination and degradation. The β-arrestin promotes the formation of the GCN2-*β*-arrestin-NEDD4L complex by binding NEDD4L to GCN2, enabling ubiquitin-mediated GCN2 degradation and thereby preventing cancer [189].

### 4.10. Melanoma

Human melanomas are the most aggressive form of malignant skin tumors, arising from neuro-ectodermal melanocytes. They can be astonishingly resistant to standard cancer therapy [191,248]. In line with this, a study showed that indole-3-carbinol (I3C) disruption of NEDD4 ubiquitination activity caused the wild-type PTEN tumor suppressor to stabilize and induced an antiproliferative response in melanoma [191,249]. An in vitro study proved that many melanoma cells show immunoreactivity for NEDD4L, and during the neoplastic transformation of melanocytes, the expression of NEDD4L might rise. Further, it was found that in xenograft models, exogenous NEDD4L expression significantly aided the growth of G-361 melanoma cells in vivo, and it was suggested that NEDD4L expression might be elevated in many melanomas to aid tumor growth [165]. Another study demonstrated that NEDD4 ubiquitinates immune checkpoint GITR and can promote melanoma cell proliferation by suppressing anti-tumor immune responses mediated by T cells [192]. A melanocytic transmembrane protein called Melan-A/MART-1 interacts with NEDD4 and ITCH and thus becomes ubiquitylated in melanoma cells. Both NEDD4 and ITCH contribute to the degeneration of the melanocyte. In pigmented cells, mutant Melan-A, which lacks ubiquitin acceptor residues, accumulates in melanosomes and has a prolonged half-life. Therefore, ubiquitylation regulates Melan-A/MART-1 lysosomal sorting and destruction from melanosomes [190]. In another study, a functional polypeptide JP1 was demonstrated to activate p-MEK1/2 and induced SP1 ubiquitination through the NEDD4L-SP1-Integrin *α*v*β*3 pathway, which inhibited melanoma cell proliferation and metastases [193]. In A375 melanoma cell lines, erastin, a ferroptosis activator, stimulates the expression of NEDD4 and FOXM1 that leads to the ubiquitination and degradation of voltage-dependent anion channels, VDAC2/3, which further leads to the suppression of erastin-induced ferroptosis in melanoma. Additionally, an in vivo study showed that NEDD4 knockdown decreased tumor size and GSH levels, and increased MDA levels in nude mice (A375) xenografts [145]. Another study reported that NEDD4 promotes K48- and K63-dependent polyubiquitination and promotes lysosomal-dependent degradation of IGPR-1, because treatment of cells with lysosomal inhibitors such as bafilomycine enhanced IGPR-1 in human skin melanoma cell lines [194].

### 4.11. Nasopharyngeal Carcinoma (NPC)

NEDD4 has been associated with the progression and development of nasopharyngeal carcinoma (NPC) [137,250]. In NPC cells, NEDD4 has tumorigenic capabilities because it promotes the EMT characteristics of cis-diamminedichloroplatinum (DDP, cisplatin) resistant cells. Overexpression of NEDD4 has been linked to EMT in DDP-resistant cells. Moreover, depletion of NEDD4 resulted in a partial reversion of EMT to MET phenotypes in resistant cells. These studies suggest that NEDD4 participates in EMT and chemoresistance in NPC cancer cells [137].

### 4.12. Neuroblastoma (NB)

Neuroblastoma is the most frequently occurring extracranial solid tumor in children, and it is defined by the neoplastic development of neural crest cells in the developing sympathetic nervous system. The original tumor can occur anywhere along the sympathetic chain, although the adrenal gland is the most common site [251,252,253]. A recent study identified NEDD4 as a novel host component required for Japanese encephalitis virus (JEV) infection of human neural cells, and it was discovered that NEDD4 enhances JEV replication by reducing autophagy. Therefore, the downregulation of NEDD4 can drastically lower JEV infectivity, allowing neuronal cells to survive [195]. A study on neuroblastoma revealed that exosomal hsa-miR199a-3p overexpression in NB can increase proliferation and migration in vitro by inhibiting NEDD4 expression, resulting in a poor prognosis [196]. NEDD4 directly binds to Myc oncoproteins and targets them for ubiquitination and degradation. Additionally, small-molecule SIRT2 inhibitors activated the NEDD4 gene, decreased the expression of N-Myc and c-Myc proteins, and inhibited the proliferation of neuroblastoma cancer cells [103].

### 4.13. Ovarian Cancer

Dysregulation of E3 ubiquitin ligases appears to be a major component in the development and maintenance of ovarian cancer chemoresistance [254,255]. In ovarian cancer tissues, NEDD4L protein expression is found to be lower than non-cancer tissues. This suggests that the downregulation of NEDD4L protein expression might contribute to the emergence of ovarian cancer [166]. For example, DNA damage-binding protein 2 (DDBP2) modulates the responsiveness of ovarian cancer cells to TGF-*β*-induced growth suppression through NEDD4L [197]. Another in vitro study in OVCAR3 cells showed that erastin or RSL3 dose-dependently induced NEDD4L expression. Moreover, NEDD4L knockdown sensitized ovarian cancer cells to erastin- or RSL3-induced cell death and tumor suppression [198].

### 4.14. Pancreatic Cancer (PCa)

Pancreatic adenocarcinoma is an aggressive cancer of the pancreas that has a poor prognosis. PCa is the 14th most common cancer in the world and the 7th greatest cause of cancer death, with around 227,000 deaths each year expected around the world [256,257,258,259,260,261]. A growing number of studies have reported that NEDD4 has been linked to the development of PCa [262]. A surfeit of studies have proven that curcumin can exhibit its anti-tumor activity in cancer cells [263]. In PCa, curcumin downregulates the NEDD4 protein and further upregulates PTEN and p73, and led to the suppression of cancer. Importantly, it was noted that suppressing NEDD4 expression resulted in decreased cell proliferation, migration, and tumor growth [120]. Regarding LTF, an iron-binding transport protein, degradation by NEDD4L prevents intracellular iron accumulation and subsequent oxidative-damage-mediated ferroptosis cell death in PANC-1 cancer cells. In addition, this study showed that erastin or RSL3 dose-dependently enhanced NEDD4L expression, and NEDD4L knockdown effectively induced cell death and erastin or RSL3 stimulated MDA production [198]. Another study suggested that by directly interacting with the NEDD4 gene core promoter and deacetylating histone H4 lysine 16, SIRT2 inhibited the expression of the NEDD4 gene. Myc oncoproteins are directly targeted by NEDD4 for ubiquitination and degradation, and it was found that SIRT2 inhibitors activate the NEDD4 gene, which results in the decreased expression of Myc proteins, and decreased the PCa cell proliferation [103]. In conclusion, NEDD4 is a possible target for the therapy of PCa.

### 4.15. Prostate Cancer

Prostate cancer is the second most observed cancer type in men and causes a series of health issues [202,264,265,266,267,268]. In the prostate gland, androgens and the androgen receptors (AR) play crucial roles for proper growth, differentiation, and physiological function. It has also been proven that AR dysregulation contributes to the advancement of prostate cancer [269,270,271,272]. In a clinical study, it was reported that the loss of NEDD4L expression was frequently observed in prostate cancer patients with more aggressive tumors. It was also shown that downregulation of NEDD4L significantly correlates with increased Gleason score, indicating the possibility of its role in prostate cancer progression [167]. A clinical study revealed that the expression of proteasomal pathway genes including PSMC4, PSMB5, and NEDD4L showed a significant upregulation. Interestingly, another member of this family, SMURF2, appears to be downregulated in organ-confined prostate tumors compared to non-organ-confined prostate tumors. Thus, dysregulation in the expression of these genes suggests a potential role in the development and spread of prostate cancer [168]. In another study, WWP2 was identified as an oncogene in prostate cancer. WWP2 influences PTEN’s degradation through a ubiquitylation-dependent pathway, regulating cellular apoptosis and increasing the development of cancer in a nude mice (DU145) xenograft, and the knockdown of WWP2 was shown to decrease tumor growth [173,199]. A negative link was discovered between HER3 and NEDD4 levels in the DU145 prostate cancer cell lines. More importantly, NEDD4 knockdown enhanced HER3 expression, making cancer cells sensitive to an anti-HER3 antibody which limited cell proliferation [172]. In conclusion, NEDD4 could be a possible target for the therapy of prostate cancer.

### 4.16. Other Cancers

Apart from these studies, the role of NEDD4 has also been studied in other cancers, such as multiple myeloma (MM), uveal melanoma, bone cancer, gall bladder cancer, etc. [156,169,273,274]. For example, NEDD4 has also been involved in the development of gallbladder carcinoma, which is an aggressive cancer with a high rate of invasiveness that continues to have a poor prognosis, with a less than 10 percentage overall survival rate [156]. A study highlighted NEDD4 as an invasion-associated molecule in gallbladder carcinoma and demonstrated that at the transcriptional level NEDD4L regulates MMP-1 (Matrix metalloproteinase-1) and MMP-13 expression, which is overexpressed at an early stage of tumor invasion in various malignant tumors [156]. Further, a study in U2OS cells reported that NEDD4L promotes the ubiquitylation of 8-Oxoguanine DNA glycosylase (OGG1), a major cellular enzyme involved in the base excision repair pathway, which plays a vital role in suppressing mutagenesis and controls genome stability [169].

## 5. Oncogenic Role of NEDD4 in Cancer

A surfeit of studies has found that NEDD4 exhibits a potential oncogenic role and is frequently overexpressed in various types of cancers. For example, in an in vivo study, it was reported that overexpression of NEDD4 enhanced the growth and metastasis of xenograft tumors, where it was abundantly expressed in colon cancer tissues and cells [109]. Similarly, the expression of NEDD4 is higher in malignant endometrium compared to benign. The levels of FOXM1, an oncogenic transcription factor, are positively correlated with NEDD4 expression in endometrial cancers. In addition, NEDD4 overexpression in endometrial cancer cells enhanced IGF-1R cell surface localization, Akt activation, and cell proliferation [155]. In GCA, NEDD4 is overexpressed, and this is linked to tumor invasion and metastasis, as well as survival rate in rats [126]. Moreover, overexpression of NEDD4 enhanced cell proliferation, reduced cell apoptosis, and promoted cell invasion and migration in bladder cancer cells. In addition, it lowered PTEN levels while increasing Notch-1 expression [105]. NEDD4 is a factor that mediates PTEN ubiquitination and inactivation, and in human NSCLC, overexpression of this protein causes a rise in the ubiquitylated form of PTEN, lowering PTEN levels and enhancing Akt activation [146]. Moreover, another study demonstrated that NEDD4 overexpression was associated with poor clinical outcomes in patients with HCC and that NEDD4 depletion inhibited proliferation, migration, and invasion of Huh7 cells through the upregulation of PTEN [111]. Interestingly, it was found that anti-tumor immunity mediated by T cells against melanoma cells is inhibited when NEDD4 is overexpressed. Moreover, the expression of NEDD4 was shown to be higher in metastatic melanoma tissues and is linked with bad prognosis [192]. Thus, these data suggest that targeting NEDD4 for cancer treatment could be a promising method.

## 6. Tumor-Suppressive Role of NEDD4

NEDD4 also exerts tumor suppressor properties in various malignancies. For example, NEDD4L prevents autophagy activity under metabolic stress by destabilizing ULK1, an autophagy protein, and ASCT2, a glutamine transporter, hence reducing mitochondrial functioning and tumor suppression [75]. Similarly, shRNA inactivation of NEDD4 increases HER3 levels in prostate cancer cells, increasing HER3 signaling and cancer cell growth [172]. Another study revealed that NEDD4 lowered the Myc protein synthesis and decreased proliferation of neuroblastoma and pancreatic cancer cells by directly binding to Myc oncoproteins and targeting them for ubiquitination and degradation [103]. Additionally, NEDD4 deregulated the PIP5K*α*-dependent PIP2 pool that promotes proliferation in BC cells via PI3K/Akt activation [113].

## 7. NEDD4 Knockout Mice

Several investigations in knockout mouse models have shown the biological activities of NEDD4. For example, an in vivo study showed decreased IGF-1 and insulin signaling, delayed embryonic development, reduced growth and body weight, and neonatal mortality in NEDD4 null mice. In addition, homozygous NEDD4 knockout in mice reduced mitogenic activity in embryonic fibroblasts and increased the amount of the adaptor protein Grb10, and mislocalized the IGF-1 receptor that is usually found on the plasma membrane. Thus, NEDD4 appears to favorably modulate IGF-1 and insulin signaling in vivo, in part through regulating Grb10 activity [275]. Consistent with this report, another study on NEDD4 null mutant mice delineated that NEDD4 deficiency results in perinatal mortality and aberrant neuromuscular structure and function. This showed that NEDD4 is essential for survival throughout mammalian embryonic development [276]. Another study showed that NEDD4 knockout in mice leads to embryonic mortality at mid-gestation, with severe heart malformations and vascular abnormalities. Further, elevated levels of thrombospondin-1 (Tsp-1), an angiogenesis inhibitor, were seen in knockout mouse embryonic fibroblasts and embryos. Interestingly, administration of aspirin (a Tsp-1 inhibitor) to pregnant heterozygote mothers resulted in lower Tsp-1 levels and a significant reduction in embryonic lethality. These findings show that NEDD4 is a Tsp-1 suppressor and that elevated Tsp-1 levels in NEDD4 knockout mice might have contributed to the developmental abnormality seen in the embryos [277]. Another study reported that NEDD4 knockout causes dendrites to become shorter and less complex, resulting in a decreased number of functional synapses and, consequently, synaptic transmission [278]. In another study, the conditional deletion of NEDD4L in lung epithelial cells led to chronic lung disease, which has many of the characteristics of IPF, such as bronchiolization and progressive fibrosis, Muc5b overexpression in honeycombing, peripheral airways, and distinctive alterations in the lung proteome [96]. Therefore, these results indicate that complete deletion of NEDD4 is lethal.

Although NEDD4 depletion does not promote tumor development on its own, it does cause tumor growth enhancement in Apc^+/min^-derived colorectal tumors, implying that NEDD4 typically reduces intestinal Wnt signaling and colonic tumorigenesis. These findings imply that NEDD4 inhibits colonic Wnt signaling and tumor growth, at least in part, by inhibiting the transcription factors LEF1 and YY1 [279]. The precise role of NEDD4 in tumorigenesis must be further validated.

## 8. NEDD4 as a Therapeutic Target

In several studies, NEDD4 has been proven to promote tumor growth, and thus targeting this protein for cancer treatment is a promising method. For example, in glioma cells, curcumin, a diarylheptanoid ingredient found in *Curcuma longa* plants, decreased proliferation, invasion, and migration by suppressing NEDD4 [125]. In addition, treatment of indole-3-carbino (I3C), a naturally occurring compound found in cruciferous vegetables, along with knockdown of NEDD4, inhibited degradation of wild-type PTEN, causing antiproliferation in melanoma cells [191]. Moreover, diosgenin, a saponin produced from the *Trigonellafoenum graecum* plant, inhibited NEDD4 expression in prostate cancer cells, resulting in anticancer action [268]. Further, Decitabine, a DNA methylation inhibitor, increased migration and invasion of gastric cancer cells by increasing the expression of NEDD4 [184]. As a result, targeting NEDD4 could be an essential therapeutic approach for the management of human cancers.

## 9. Conclusions

By combining studies, it is clear that NEDD4/NEDD4-like E3 ligases are crucial to the multistep process that causes different forms of cancer. In most cancer types, NEDD4 inhibits tumorigenesis by increasing the degradation of NEDD4 substrates which have a crucial oncogenic role in various malignancies; however, its significance in a few cancer types is still debated. Therefore, due to its role in tumorigenesis, NEDD4 can be a potential target in drug discovery against various types of cancer. Because of NEDD4’s many substrates and dual functions, treatment approaches that disrupt NEDD4’s interactions with these substrates with no/fewer side effects might be more suitable than those that directly target NEDD4 activity. However, further studies are required to completely understand the relevance of NEDD4 in tumorigenesis.

## Figures and Tables

**Table 1:** Regulation of NEDD4-like E3 ligases in various cancers in clinical studies.

Cancer Type	Tissues	Results	Reference
Breast Cancer	BC tissues	↑WWP1 ^a^	[150]
	BC tissues	↑NEDD4, P34^SEI-1^	[151]
	BC tissues	↑WWP1	[152]
	BC tissues	↑NEDD4	[54]
Colorectal Cancer	CRC tissues	↑NEDD4	[153]
	CRC tissues	↑NEDD4 ↓NEDD4L	[76]
	CRC tissues	↑NEDD4	[109]
Endometrial Cancer	EC tissues	↓NEDD4L	[154]
	EC tissues	↑NEDD4, FoxM1	[155]
Gall bladder Cancer	Gallbladder cancer tissues	↑NEDD4L, MMP-1, MMP-13	[156]
Gastric Cancer	Gastric cancer tissues	↑NEDD4	[153]
	Gastric cancer tissues	↓NEDD4L	[157]
	Gastric cancer tissues	↑NEDD4	[126]
	Gastric cancer tissues	↓NEDD4L ↑HIF-1*α*	[158]
Glioma/Glioblastoma	Glioma tissues	↓NEDD4L	[159]
	Glioma tissues	↑NEDD4 ↓CNrasGEF	[160]
	Glioma tissues	↑NEDD4	[161]
	Glioma tissues	↑SMURF1	[162]
Liver Cancer	HCC tissues	↑NEDD4, p-Akt ↓PTEN	[111]
	HCC tissues	↓NEDD4L	[163]
Lung Adenocarcinoma	NSCLC tissues	↑NEDD4 ↓PTEN	[97]
	Lung adenocarcinoma tissues	↑NEDD4, p-Akt ↓PTEN	[121]
	NSCLC tissues	↓NEDD4L	[164]
Melanoma	Melanoma tissues	↑NEDD4L	[165]
Ovarian Cancer	Ovarian cancer tissues	↓NEDD4L	[166]
Prostate Cancer	PC tissues	↓NEDD4L	[167]
	PC tissues	↑NEDD4L, PSMB5, PSMC4	[168]

↑—Increase/upregulation; ↓—decrease/downregulation, ^a^—Differential expression from Category I to IV.

**Table 2:** Mechanistic role of NEDD4-like E3 ligase family in various cancers.

Cancer Type	In Vitro/ In Vivo	Model/Cell Lines	NEDD4 Family	Mechanism of Action/Outcomes	Reference
Bladder Carcinoma	In vitro	RT4 cells	NEDD4 (KD)	↓Cell proliferation, Viability, Migration, Invasion, Notch-1 ↑PTEN, Apoptosis	[105]
			NEDD4 (OE)	↑Cell proliferation, Invasion, Migration, Notch-1 ↓Apoptosis, PTEN	
Bone Cancer	In vitro	U2OS cells	NEDD4L (KD)	↑OGG1	[169]
Breast Cancer	In vitro	MCF-7, T47D	WWP1 (KD)	↓Cell growth, Colony formation	[150]
	In vitro	MDA-MB-231	NEDD4 (KD)	↓NEDD4 ↑MKP3	[170]
	In vitro	MCF-7/T47D	WWP1 (KD)	↑LATS1 ↓Cell number	[171]
			NEDD4, ITCH (KD)	↑LATS1	
			SMURF1 (KD)	↓LATS1	
	In vitro	MCF-7	NEDD4 (KD) NEDD4 (KD) + NRG-1	↑HER3 ↑pAkt, pERK/2, Cell proliferation, Colony number, pHER	[172]
	In vivo	MCF-7 (shNEDD4) BALB/c- nu/nu mice xenograft	NEDD4 (KD) + NRG-1 + HER3 mAb	↓Tumor volume	[172]
	In vitro	MDA-MB-231	WWP2 (KD)	↓WWP2, pAkt (S473), Cell proliferation, Colony number ↑Cdh1	[173]
	In vitro	MDA-MB-231 (shPIPKI*γ*i5)	NEDD4 (KD)	↑Mig6	[174]
	In vitro	MDA-MB-231, MCF-7	NEDD4 (KD)	↓ITG *β*4 ubiquitination	[175]
	In vitro	MCF-7 (sh-NEDD4)	NEDD4 (KD) + Estradiol	↓ER, HER3 ↑Cell proliferation	[176]
	In vitro	SKBR3	NEDD4 (OE)	↓PIP5K*α*	[113]
	In vitro	MCF-7, BT474	NEDD4 (Ac) (fulvestrant)	↓Cx43	[177]
	In vitro	MDA-MB-231, T47D, BT549, ZR-75-1, MCF-7	NEDD4 (KD)	↓Cell growth	[54]
		MDA-MB-231, T47D	NEDD4 (KD)	↓IGF-1R, p-Akt^Ser473^ ↑PTEN	
	In vitro	MDA-MB-231, MDA-MB-436	NEDD4 (KD)	↓Cell proliferation, Migration, Mammosphere formation, ALDH1A, CD44	[178]
	In vitro	MDA-MB-231 (PRRG4-OE)	NEDD4 (KD)	↑Robo1	[124]
		MDA-MB-231 (PRRG4-OE), HCC1954	NEDD4 (KD)	↓Cell migration, Invasion	
	In vitro	SKBR3	SMURF1 (KD)	↑HER2	[179]
			NEDD4 (KD)	↑SMURF1	
	In vitro	MCF-7	NEDD4 (In) by RF-A	↑VDAC2, apoptosis, Ferroptosis ↓Cell viability	[180]
Cervical Cancer	In vitro	HeLa cells	NEDD4 (OE)	↓PTEN, PTEN-induced apoptosis	[181]
	In vitro	HeLa cells	NEDD4 (KD)	↑Beclin 1	[119]
	In vitro	HeLa cells	NEDD4L (OE)	↓ULK1	[182]
			NEDD4L (KD)	↑ULK1	
	In vitro	HeLa-Cx43 cells	NEDD4 (KD)	↑Cx43	[93]
		HeLa-CCL2 cells	NEDD4 (OE)	↓Cx43	
		C33A cells	NEDD4 (OE)	↓Gap junction, Cx43	
Colorectal Cancer	In vitro	HCT-15, LoVo	NEDD4 (KD)	↑Cell morphological alterations, Reorganization of the actin cytoskeleton ↓Cell growth	[102]
	In vitro	SW1116	NEDD4 (KD)	↑p21, NDRG1	[183]
	In vitro	LoVo	NEDD4 (OE)	↑Cell growth, Vimentin, N-cadherin, snail, ATF-1 ↓E-cadherin, FOXA1, miR-340	[109]
			NEDD4 (KD)	↑ FOXA1	
		Caco-2	NEDD4 (KD)	↓Cell growth, Cell proliferation, Colony number, Vimentin, N-cadherin, Snail, ATF-1 ↑Apoptosis, Cyto C, PUMA, Apaf-1, Bax, E-cadherin, FOXA1, miR-340	
			NEDD4 (OE)	↑Cell proliferation, Colony number ↓Apoptosis, Cyto C, PUMA, Apaf-1, Bax, FOXA1	
Endometrial Cancer	In vitro	Ishikawa	NEDD4 (OE)	↑Cell growth, p-ERK, pAkt, IGF-1R	[155]
Gall bladder Cancer	In vitro	TGBC1TKB	NEDD4 (KD)	↓Invasion, MMP-1, MMP-13	[156]
Gastric Cancer	In vitro	AGS, N87	NEDD4 (KD)	↓Cell number, Migration, Invasion	[126]
	In vitro	MGC803	NEDD4 (Ac)	↑NEDD4, Migration, Invasion	[184]
Glioma/Glioblastoma	In vitro	NHA-E6/E7/hTERT	NEDD4 (KD)	↑PTEN	[185]
	In vitro	U251	NEDD4 (KD)	↓Migration, Invasion	[160]
			NEDD4 (OE)	↑Migration, Invasion	
	In vitro	U251, U87	NEDD4 (KD)	↓Migration, Invasion	[161]
			NEDD4 (OE)	↑Migration, Invasion ↓Rap2a	
	In vitro	A1207, SNB19	NEDD4 (In)	↓p-Akt, Notch-1, Migration, Invasion	[125]
			NEDD4 (OE)	↓Apoptosis ↑Migration, Invasion	
			NEDD4 (KD)	↑Apoptosis ↓Migration, Invasion, Notch-1, p-Akt	
	In vitro	U87-MG	SMURF1 (KD)	↓Migration, Invasion, Vimentin, MDM2 ↑E-cadherin, p53, Cleaved Caspase-3, Cleaved PARP	[162]
	In vitro	U87-MG, M059K	NEDD4L (OE)	↓Cell viability ↑p-*β*-catenin	[186]
		U87-MG	NEDD4L (OE)	↓*β*-catenin, Cyclin-D1	
Liver Cancer	In vitro	HepG2 cells	NEDD4 (OE)	↓GUCD1	[187]
	In vitro	Huh7 cells	NEDD4 (KD)	↓Cell proliferation, Migration, Invasion, p-ERK1/2, p-Akt, p-STAT3 ↑Cytoskeletal changes, S phase cell cycle arrest, PTEN	[110]
	In vitro	Huh7, Hep3B, PLC/PRF/5, SMMC7721, LO2	NEDD4 (KD)	↑PTEN, E-cadherin ↓Cell proliferation, Migration, p-Akt, Vimentin	[111]
	In vitro	SK-hep1 HCCLM3	NEDD4 (KD) NEDD4 (OE)	↑Cell number, p-ERK1/2 ↓ Cleaved caspase 3 ↑BIRC3, CASP2, CASP7	[163]
	In vivo	Nude mice (HCCLM3-NEDD4L)	NEDD4L (OE)	↓Tumor weight ↑p-ERK1/2	[163]
	In vitro	QGY7703, SMMC7721	NEDD4 (KD)	↓NEDD4, Cell proliferation, Cell viability, Migration, Invasion, p-Akt ↑Apoptosis, LATS1	[106]
			NEDD4 (OE)	↑NEDD4, Cell proliferation, Cell viability, Migration, Invasion, p-Akt ↓Apoptosis, LATS1	
Lung Cancer	In vitro	NCI-H460	NEDD4 (OE)	↓PTEN ↑p-Akt, Cell growth	[97]
		NCI-H292	NEDD4 (KD)	↑PTEN, p21, Gelsolin ↓p-Akt, c-Myc, Cell growth	
	In vivo	Nude mice (NCI-H292-shNEDD4) xenografts	NEDD4 (KD)	↓Tumor volume, p-Akt ↑PTEN	[97]
		Nude mice (NCI-H460-NEDD4-HA) xenografts	NEDD4 (OE)	↑Tumor volume, p-Akt ↓PTEN	
	In vitro	A549	NEDD4 (KD)	Transport of TMEPAI to the lysosome	[188]
	In vitro	A549	NEDD4 (KD)	↑GCN2	[189]
	In vitro	A549	NEDD4L (KD)	↑pSMAD2	[139]
	In vitro	HCC827/ER cells + erlotinib	NEDD4 (KD)	↑PTEN ↓p-Akt	[146]
			NEDD4 (OE)	↓PTEN ↑p-Akt	
		H1650/ER cells + erlotinib	NEDD4 (KD) + PTEN(OE)	↑PTEN	
	In vivo	Nude mice (HCC827/ER cells) xenograft	NEDD4 (KD)	↓Tumor growth, Tumor weight	[146]
	In vitro	A549	NEDD4 (KD)	↓NEDD4, EGF stimulated migration, EGF stimulated cathepsin expression	[122]
			NEDD4 (KD) + NEDD4	↑EGF stimulated migration	
	In vitro	A549	NEDD4 (KD)	↓NEDD4, Cell proliferation, Migration, Invasion, p-Akt, NF-kB, mTOR ↑BAD, PTEN	[121]
	In vitro	H1975, HCC827	NEDD4L (KD)	↑Cell proliferation, Migration, Invasion, Cell number	[164]
	In vivo	Nude mice (HCC827-LUC-shNEDD4) xenografts	NEDD4L (KD)	↑Tumor growth, Metastasis	[164]
Melanoma	In vitro	Melan-A transduced SK-Mel-37 cells	NEDD4, ITCH (KD)	↑Melan-A	[190]
			NEDD4, ITCH (OE)	↓Melan-A	
	In vitro	G-361 + I3C	NEDD4 (KD)	↑PTEN, MDM2 ↓p-MDM2, Bcl-2, Caspase 3	[191]
	In vitro	G-361	NEDD4L (KD)	↓NEDD4L, Cell growth	[165]
	In vivo	BALB/c nude mice (A2058) xenograft	NEDD4L (OE)	↑Tumor volume	[165]
	In vitro	Jurkat cells	NEDD4 (OE)	↓GITR ↑Foxp3, IL-2	[192]
			NEDD4 (KD)	↑GITR ↓Foxp3, IL-2	
		A375 + Jurkat cells	NEDD4 (OE)	↑Cell survival rate, IL-2, Foxp3 ↓GITR	
	In vitro	A375	NEDD4L (OE)	↓Cell proliferation, Migration, SP-1	[193]
	In vitro	A375 + erastin	Wt-NEDD4 (OE)	↓VDAC2/3 protein level ↑K48-linked ubiquitination of VDAC2/3	[145]
			NEDD4 (KD)	↑VDAC2/3	
		A375 and G361	NEDD4 (KD)	↑Erastin-induced cell death, ROS production, Iron accumulation, GSH depletion, GSSG generation	
			NEDD4 (OE)	↑Cell viability	
	In vivo	Nude mice (A375) xenografts	NEDD4 (KD)	↓Tumor size, GSH levels ↑MDA levels	[145]
	In vitro	A375	NEDD4 (KD)	↓NEDD4 ↑IGPR-1	[194]
		SK-MEL-28	NEDD4 (OE)	↓IGPR-1	
Nasopharyngeal Carcinoma	In vitro	CNE1-DDP, CNE2-DDP	NEDD4 (KD)	↑MET, E-cadherin, ZO-1 ↓Cell attachment and detachment capacity, Migration, Invasion, N-cadherin, Vimentin, Slug	[137]
Neuroblastoma	In vitro	BE (2)-C	NEDD4 (KD)	↑N-Myc, c-Myc protein	[103]
	In vitro	SK-N-SH	NEDD4 (KD)	↓NEDD4 mRNA, JEV infection ↑LC3-II, Beclin 1	[195]
			NEDD4 (OE)	↑JEV NS3 expression	
	In vitro	SK-N-SH	NEDD4 (OE)	↓Cell proliferation, Migration	[196]
			NEDD4 (OE)+ hsa-miR199a-3p mimic	↑Cell proliferation, Migration	
Ovarian Cancer	In vitro	CP70, CP70-DDB2, PEO1	NEDD4L, DDB2 (KD)	↑p-Smad2	[197]
		CP70	NEDD4L, DDB2 (OE)+ TGF-*β*	↑p-Smad2	
	In vitro	OVCAR3	NEDD4 (KD)	↓NEDD4	[198]
			NEDD4 (KD) + RSL3	↑Cell death, MDA, Oxidative stress, LTF protein expression	
			NEDD4 (KD) + Erastin	↑Cell death, MDA, Oxidative stress, LTF protein expression	
Pancreatic Cancer	In vitro	MiaPaca-2	NEDD4 (KD)	↓NEDD4 ↑N-Myc, c-Myc protein, SIRT2	[103]
	In vitro	Patu8988, Panc-1	NEDD4 (OE) + Curcumin	↑Cell proliferation, Migration, Invasion ↓Apoptosis, PTEN, p73	[120]
			NEDD4 (KD) + Curcumin	↓Cell growth, Migration, Invasion ↑Apoptosis, PTEN, p73	
	In vitro	Panc-1	NEDD4 (KD)	↓NEDD4	[198]
			NEDD4 (KD) + RSL3	↑Cell death, MDA, Oxidative stress, LTF protein expression	
			NEDD4 (KD) + Erastin	↑Cell death, MDA, Oxidative stress, LTF protein expression	
Prostate Cancer	In vitro	DU145	WWP2 (KD)	↑PTEN ↓p-Akt, Cell proliferation	[199]
			WWP2 (KD) + Doxorubicin	↑Apoptosis	
	In vivo	Nude mice (DU145-shWWP2) xenograft	WWP2 (KD)	↓Tumor growth	[199]
	In vitro	DU145	NEDD4 (KD) + NRG-1	↑Cell proliferation, Migration, p-HER3, p-Akt1, Spheroid formations, HER3	[172]

↑—Increase/upregulation; ↓—decrease/downregulation, OE—overexpression, KD—knockdown, Ac—activation, In—inhibition.

## Data Availability

Not applicable.

## Abbreviations

AGE—Advanced glycation end products; *ARRDC3*—arrestin domain-containing 3; ASCT2—alanine-serine-cysteine transporter 2; ATF1—Activating Transcription Factor 1; Cdh1—cadherin1; CNrasGEF—cyclic nucleotide ras guanine nucleotide exchange factor; Cx43—connexin 43; DDP—cisplatin; DUBs—deubiquitinases; DVL2—disheveled segment polarity protein 2; EGFR—epidermal growth factor receptor; EMT—epithelial–mesenchymal transition; ENaC—epithelial sodium channel; ER—estrogen receptor; EVs—extracellular vesicles; EZH2—enhancer of zeste homolog 2; FAK—focal adhesion kinase; FGFRs—fibroblast growth factor receptors; FOXM1—forkhead box protein M1; GCA—gastric cardia adenocarcinoma; GCN2—general control nonderepressible 2; GITR—glucocorticoid-induced TNFR-related protein; *GUCD1*—guanylyl cyclase domain-containing 1; HDAC5—histone deacetylase 5; HECT—homologous to E6-AP C-terminus; IGF-1—insulin-like growth factor I; IGPR1—immunoglobulin and proline-rich receptor-1; IRS2—Insulin Receptor Substrate 2; I3C—indole-3-carbinol; ITG*β*4—integrin, *β4*; ITCH—Itchy E3 Ubiquitin–Protein Ligase; JEV—Japanese encephalitis virus; LAPTM5—lysosomal-associated protein transmembrane 5; LATS1—large tumor suppressor kinase 1; LGR5—leucine-rich repeat-containing G-protein coupled receptor 5; LUAD—lung adenocarcinoma; MAP—mitogen-activated protein; MAPK—mitogen-activated protein kinase; Mdm2—mouse double minute2 homologue; Melan-A—melanoma antigen; MET—mesenchymal–epithelial transition; MHC—Major Histocompatibility Complex; MMP-1—matrix metalloproteinase-1; N4BP1—NEDD4 Binding Protein 1; NDRG1—N-Myc downstream regulated gene-1; NEDD4—neuronal precursor cell-expressed developmentally downregulated 4; NEDD4L—neuronal precursor cell-expressed developmentally downregulated 4-like; NEDL1/2—NEDD4-like ubiquitin–protein ligase-1/2; NF-κB—nuclear factor kappa B; OGG1-8—oxoguanine glycosylase; PI3K—phosphoinositide 3-kinase; PIPKI*γ*i5—type I *γ* phosphatidylinositol phosphate 5-kinase i5; PIP5K*α*—phosphatidylinositol-4-phosphate 5-kinase *α*; PKCδ—protein kinase C *δ;* PMEPA1—Prostate Transmembrane Protein, Androgen-Induced 1; PRRG4—proline-rich *γ*-carboxyglutamic acid protein 4; PTEN—phosphate and tensin homologue; PTMs—post-translational modifications; RFS—recurrence-free survival; RING—really interesting new gene; RIP—receptor-interacting protein; Robo1—Roundabout Guidance Receptor 1; SIRT2—Sirtuin 2; SMURF1/2—SMAD ubiquitination regulatory factors 1 and 2; STAT3—Signal Transducer and Activator of Transcription 3; TGF-*β*—transforming growth factor-*β*; TMEPAI—transmembrane prostate androgen-induced protein; TMZ—temozolomide; TNIP1—TNFAIP3 Interacting Protein 1; ULK1—Unc-51 like autophagy activating kinase 1; VDAC2/3—voltage-dependent anion-selective channel protein 2/3; VEGFR2—vascular endothelial growth factor receptor-2; *VPS34**—*vacuolar protein sorting 34; WBP2—WW Domain-Binding Protein 2; WBP2NL—WBP2 N-terminal-like; WWP1/2—WW Domain-Containing E3 Ubiquitin–Protein Ligase 1/2.
